# Clinical and Radiographic Outcomes of Anterior Lumbar Interbody Fusion With Anterior Plate Fixation

**DOI:** 10.7759/cureus.55139

**Published:** 2024-02-28

**Authors:** Jacob Razzouk, Daniel Cheng, Davis Carter, Shaurya Mehta, Omar Ramos, Wayne Cheng

**Affiliations:** 1 Orthopaedic Surgery, School of Medicine, Loma Linda University, Loma Linda, USA; 2 Biological Sciences, University of Southern California, Los Angeles, USA; 3 Orthopaedics, School of Medicine, University of California Riverside, Riverside, USA; 4 Spine Surgery, Twin Cities Spine Center, Minneapolis, USA; 5 Orthopaedic Surgery, Jerry L. Pettis VA Medical Center, Loma Linda, USA

**Keywords:** odi, vas, outcomes, radiographic, anterior lumbar plating, anterior lumbar interbody fusion

## Abstract

Background: Reports on the outcomes following instrumented anterior lumbar interbody fusion (ALIF) with anterior plate fixation are limited. The aim of this study was to assess the clinical and radiographic outcomes of patients undergoing ALIF with anterior plate fixation.

Methods: Medical records and radiographic imaging were reviewed for 100 patients who received ALIF with anterior plate fixation between 2008 to 2021 and completed at least one year of follow-up and postoperative imaging. Prospectively collected patient data included indication for surgery, BMI, age, number and location of levels treated, complications, reoperation rates, fusion rate, and measurements of global lordosis, disc space height, and segmental angulation.

Results: A total of 100 patients were included in this study. The mean length of follow-up was 81.37 months. Changes in preoperative-to-postoperative Oswestry Disability Index (ODI) and Visual Analog Score (VAS) demonstrated improvements of 30.86% and 18.56%, respectively. Major vascular injuries occurred in 3% of the cases. A hardware failure rate of 5% was observed. The reoperation rate was 10%. The radiographic fusion rate derived from computed tomography (CT) and plain film was 95.45% and 88.87%, respectively. Postoperative global lordosis demonstrated a 6.08% increase. Postoperative segmental angulation measurements increased by 57.74%. No statistically significant differences in clinical or radiographic outcomes were observed based on the spine level of fusion or the number of fusions performed.

Conclusions: ALIF with anterior plate fixation achieved an adequate fusion rate with improvements in ODI and VAS scores meeting minimal clinically important difference thresholds. Postoperative disc space heights demonstrated significant improvements compared to preoperative measurements.

## Introduction

Over 350,000 patients in the United States receive an interbody spinal fusion, which is the most common spinal procedure performed in the U.S. with an increase in volume of 62.3% from 2004 to 2015 [1]. Specific to anterior lumbar interbody fusion (ALIF), Varshneya et al. identified a 68.5% increase in ALIF procedures performed in the U.S. from 2007 to 2014 [2]. Over 35% of ALIF procedures are performed due to a primary diagnosis of degenerative disc disease, 34.5% involve multilevel fusion, and 80.1% of all ALIF procedures involve spinal instrumentation [2]. Prior to the twentieth century, achieving an instrumented anterior fusion approach was not possible. However, in the early 2000s, instrumented ALIF became feasible due to advancements in screw-locking systems to prevent backing out. A further innovation in the ALIF procedure has been the introduction of anterior plating systems to provide additional anterior stabilization [3,4]. Nevertheless, due to the heterogeneity of implants and levels studied, more investigation is needed to assess the effects of ALIF with anterior plating [5-8]. 

Both clinical and radiographic outcomes following ALIF are important to consider in the evaluation and management of patients postoperatively [9]. Plain static radiographs and computed tomography (CT) are commonly used for the assessment of postoperative fusion status and disc space measurements [9-11]. The purpose of this study was twofold: to report the clinical outcomes of ALIF with anterior plating system for treatment of degenerative disc disease, and to compare preoperative and postoperative plain film and CT radiographic measurements of global lumbar lordosis, disc space height, segmental angulation, and extent of radiographic fusion. 

## Materials and methods

Data collection 

Following IRB approval (#5220032), medical and plain film radiographic records were retrieved for 100 patients who underwent single-level or multilevel ALIF with anterior plating (Aegis plate, DePuy Synthes, Raynham, MA) between the years 2008 and 2021. Criteria for inclusion in this study were defined as patients with at least a 12-month follow-up period as well as at least 12 months of available postoperative imaging. Patients with spinal neoplasm or infection, below 18 or above 85 years of age, or without at least 12 months of postoperative follow-up and radiographic imaging were excluded from review. Prospectively collected clinical data included length of follow-up, preoperative diagnosis, age, body mass index (BMI), number of treated levels, preoperative and postoperative Visual Analog Score (VAS) and Oswestry Disability Index (ODI), instances of intraoperative or postoperative complications, malfunctions, or complications related to the device or procedure, and the need and reason for revision after the index surgery. Minimum clinically important difference (MCID) was defined as a ≥ 30% change between preoperative and postoperative ODI scores and a 1.2-point change between preoperative and postoperative VAS scores [12-16]. 

Radiographic assessment 

**Figure 1:**
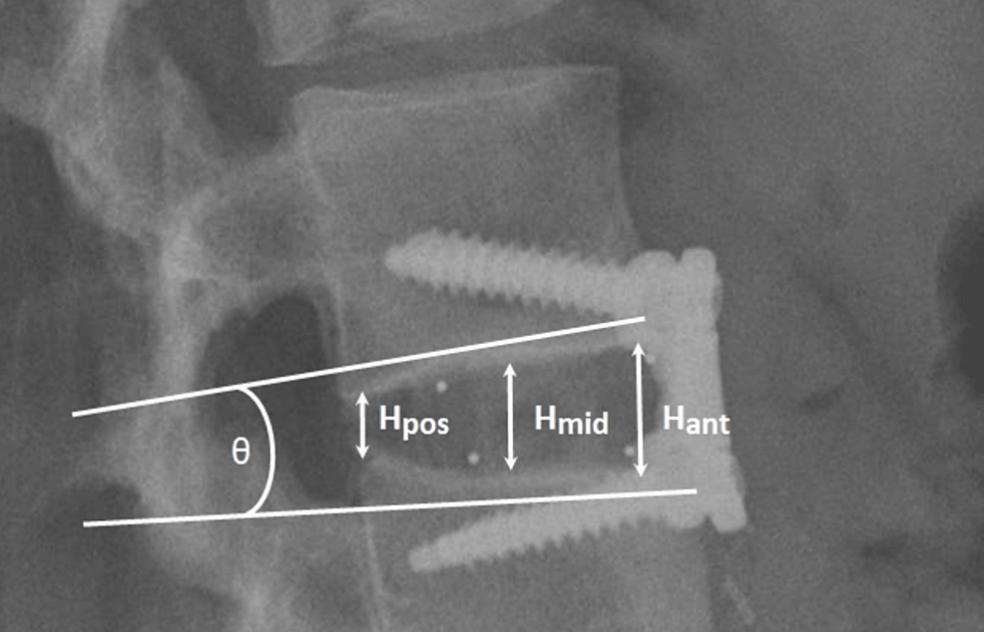
Radiographic Measurement of Intervertebral Disc Space Height and Segmental Angulation. Anterior disc space height (Hant) was measured as the vertical distance between the anterior inferior vertebral endplate of the superior vertebral body and the anterior superior vertebral endplate of the inferior vertebral body. Middle disc space height (Hmid) and posterior disc space height (Hpos) were similarly measured using the middle and posterior vertebral endplates. Segmental angulation was measured as the degree of angulation (θ) formed between the endplates of the superior and inferior vertebral bodies.

Preoperatively, patients received lumbar and full-spine anteroposterior (AP) and standing neutral lateral radiographs in a standardized standing position to exclude scoliosis and to measure spino-pelvic parameters. Radiographic imaging was evaluated by a blinded, independent reviewer for several parameters using the IMPAX6 picture archiving and communications system (Agfa-Gavaert, Mortsel, Belgium). These radiographic parameters were the following preoperative and postoperative measurements: global lordosis from L1-L5, segmental angulation, the extent of postoperative radiographic fusion, and the anterior, middle, and posterior disc space height. Segmental angulation and anterior, middle, and posterior intervertebral disc height measurements were recorded using standing neutral lateral radiographs (see Figure 1) [11,17,18]. The extent of postoperative radiographic fusion was assessed using CT and/or plain film imaging, with the fusion rate graded on the Cook et al. scale [19]. The presence of fusion was defined as evidence of bridging bone connecting the adjacent vertebral bodies and an absence of radiolucent lines greater than 50% of the implant site [11]. Global lordosis from L1-L5 was measured as the degree of angulation formed between the superior endplate of the L1 vertebral body and the inferior endplate of the L5 vertebral body. 

Statistical analysis 

All statistical analyses were performed using SPSS v. 28 (IBM Corp., Armonk, NY). Descriptive summary statistics for all clinical and radiographic data utilized means and standard deviations for all continuous data, and frequency counts and percentages for all categorical data. Paired sample t-tests were conducted to determine differences between preoperative and postoperative clinical and radiographic continuous variables. A paired sample t-test was also employed to evaluate for differences between plain film and CT-derived measurements of fusion status. Sex differences for all continuous variables were assessed using independent sample t-tests with Levene’s test for equality of variances. Independent sample t-tests and one-way analysis of variance (ANOVA) with posthoc Bonferroni and Tukey corrections were employed to evaluate for differences in fusion rate across levels. All statistical analyses were two-tailed with an alpha setting of 0.05 denoting statistical significance. 

## Results

Clinical outcomes 

A total of 64 female and 36 male patients were included in this study. Of the total 100 patients, 79 received a single-level fusion, 20 received a two-level fusion, and one patient received a three-level fusion, for a total of 122 ALIF procedures performed. Per level, two procedures were conducted at L2-3, eight fusions were performed at L3-4, 40 fusions were performed at L4-5, and 72 fusions were performed at L5-S1. rhBMP-2 was used for all procedures. 

The mean patient age at the time of surgery was 50.66 ± 13.33 years. The mean length of follow-up was 81.37 ± 30.58 months, and the mean patient BMI was 29.26 ± 6.91 kg/m2. Mean preoperative ODI and VAS scores were 52.63 and 6.52, respectively. Mean postoperative ODI and VAS scores were 36.38 and 5.31, representing score decreases of 16.25 (30.86%) and 1.21 (18.56%) compared to baseline preoperative scores. The major vascular injury rate was 3%. No postoperative infections were encountered. A hardware failure rate of 5% was observed. The reoperation rate was 10%, with device malfunction events described in 50% of all incidents of reoperation. No sex differences were observed with respect to any clinical or radiographic outcomes. No statistically significant differences in clinical or radiographic outcomes were observed based on disc level of fusion (L2-3, L3-4, L4-5, or L5-S1) or number of fusions performed (single- versus multi-level). 

Radiographic outcomes 

Among the fusion procedures with available CT imaging for assessment (n = 56), the mean radiographic fusion rate as derived from CT was 95.45% ± 19.00%. Among the fusions with available plain film imaging for assessment (n = 128), the mean radiographic fusion rate as assessed using plain film imaging was 88.87% ± 21.46%. When comparing plain film and CT-derived fusion rates among patients with both forms of imaging available (n = 56), a significant difference in radiographic fusion rate was not observed between modalities (p = 0.289). With respect to mean anterior, middle, and posterior intervertebral disc height, postoperative measurements demonstrated increases of 70.36%, 51.78%, and 51.45% compared to baseline preoperative measures. Mean postoperative global lordosis demonstrated a 6.08% increase compared to preoperative global lordosis. Mean postoperative segmental angulation measurements increased by 57.74% compared to preoperative angulation values. Table 1 details the mean preoperative and postoperative radiographic measurements with respect to global lordosis, segmental angulation, and intervertebral disc height at the anterior, middle, and posterior sections of the disc space. 

**Table 1:** Comparison of Preoperative and Postoperative Radiographic Measurements

Comparison of Preoperative and Postoperative Radiographic Measurements				
Radiographic measure	Preoperative	Postoperative	Mean difference	p-value
Global lordosis	47.67°	50.57°	2.9°	0.092
Segmental angulation	8.69°	15.05°	6.36°	< .001>
Anterior disc space height	9.38 mm	15.98 mm	6.60 mm	< .001>
Middle disc space height	7.57 mm	11.49 mm	3.92 mm	< .001>
Posterior disc space height	4.82 mm	7.30 mm	2.48 mm	< .001>

## Discussion

This study contributes to the literature by reporting the clinical and radiographic outcomes of 100 patients who underwent single-level or multilevel ALIF with anterior plate fixation. Assessing radiographic fusion rate using CT and plain film imaging, our study was also rigorous in its detailing with respect to evaluating anterior, middle, and posterior disc space height, and differences in radiographic and clinical measurements with respect to disc level and number of levels fused. 

Clinical outcomes 

Regarding the reoperation rate, our observed reoperation rate of 10% was within the range reported in the existing literature. Irmola et al. and Deyo et al. reported rates for lumbar spinal fusion ranging from 9.8% to 12.5% [20,21]. Previous literature has demonstrated vascular injury rates ranging from 1.9% to 4.6% [22-25]. We observed a 3% rate of major vascular injury. 

While our study only observed mean improvements in ODI and VAS scores of 16.25 and 1.21, respectively, both the ODI and VAS patient scores in this study met MCID parameters. In the literature, Szadkowski et al. found lower back and leg VAS score improvements of 2.4 and 1.7 and mean ODI improvement of 23.3 points following standalone ALIF at L5-S1 using anterior plate fixation, while Norotte and Barrios demonstrated a mean ODI improvement of 39 points after a two-year follow-up [26,27]. Our more modest findings may be attributed to the fact that our study conducted a longer follow-up period with an average of 81.37 ± 30.58 months. 

Radiographic outcomes 

Radiographic findings demonstrated statistically significant differences in preoperative versus postoperative measurements with respect to segmental angulation as well as anterior, middle, and posterior intervertebral disc height. While the literature is sparse with respect to measurements of the middle intervertebral disc height, our findings with respect to anterior and posterior disc height are concurrent with the literature [28-31]. Matsumura et al. observed significant differences in preoperative versus postoperative disc space height with mean differences of 10.0 mm and 6.5 mm for anterior and posterior measurements, respectively. Matsumura et al. also evaluated segmental angulation and reported a mean preoperative-to-postoperative difference of 7.3°. Evaluating standalone ALIF among patients with degenerative disc disease and two years of follow-up, Lammli et al. observed significant differences in preoperative versus postoperative disc space height ranging from 2.84 - 3.74 mm. The values demonstrated in our study are in between those of Matsumura et al. and Lammli et al. as our observed values were 6.36° for segmental angulation, and 6.60 mm, 3.92 mm, and 2.48 mm for anterior, middle, and postoperative disc heights, respectively. However, our values are similar compared to those reported in the systematic review of standalone ALIF outcomes by Giang et al., which reported mean values of 7.9° for segmental angulation, 7.1 mm for anterior disc height, and 2.8 mm for posterior disc height [32]. 

Our study is unique in that we evaluated the effects of disc level and the number of levels fused on clinical and radiographic outcomes. While future multicenter research will be required to further explore this topic, our initial findings did not observe any statistically significant differences (positive or negative) in clinical or radiographic outcomes based on disc level or number of levels fused. 

Regarding the radiographic fusion rate, our study utilized both plain film and CT imaging modalities for the assessment of fusion. Fusion assessment is difficult to compare across clinical studies due to heterogeneity in definitions of fusion criteria, levels fused, and devices utilized intraoperatively [33]. While some studies require bridging bone and no motion in lateral flexion and extension radiographs, others allow up to 5° of vertebral motion [19]. A systematic review by Manzur et al. demonstrated that the rate of fusion for standalone anterior lumbar interbody fusion is generally high, though the exact success rate varies according to factors such as the surgical approach, implants, and bone graft options utilized intraoperatively [34]. The cumulative effect of these factors can result in wide variations in the reported incidence of bony fusion. Nevertheless, our observed fusion rate as derived from either CT or plain film was higher than the rate of 79.8% reported in the systematic review by Giang et al [32]. Furthermore, our study is unique in that we analyzed the rate of fusion based on both CT and plain film radiographic imaging and compared the derived rates from both modalities. Our consistent usage of rhBMP-2 may contribute to the high fusion rate observed in our study compared to other reports. 

Limitations 

Our study is not without several limitations. The retrospective, chart-based nature of our data collection could limit the completeness of documentation in our study and potentially introduce underreporting of minor complications such as superficial wound infections or hematomas. In addition, this study may not have been sufficiently powered when conducting subanalyses including per disc level and per number of levels fused. Future research and meta-analyses will be required to fully understand these subgroups. Finally, regarding global lordosis, we observed a mean increase in global lordosis of 6.08%. While this rate is consistent with the current literature, nevertheless, this finding was not sufficiently powered to denote statistical significance [7,35]. 

## Conclusions

This study reports the clinical and radiographic outcomes of 100 patients undergoing single-level or multilevel anterior lumbar interbody fusion with anterior plate fixation. The study observed a fusion rate of 95.45% as assessed on CT imaging, and a rate of 88.87% derived from plain film imaging. ALIF with anterior plate fixation demonstrates adequate MCID improvements in ODI and VAS scores. The reoperation rate was 10%, with device malfunction events described in 50% of all incidents of reoperation. Five patients experienced hardware failure. Around 3% of patients experienced a major vascular injury. With respect to mean anterior, middle, and posterior intervertebral disc height, postoperative measurements demonstrated increases of 70.36%, 51.78%, and 51.45% compared to baseline preoperative measures. Mean postoperative segmental angulation measurements increased by 57.74% compared to preoperative angulation values.
